# Exercise-mimicking effects of betaine in chronic disease prevention and management

**DOI:** 10.3389/fnut.2025.1762908

**Published:** 2026-01-13

**Authors:** Yuhui Xu, Jianhong Gao, Minghui Wang, Hu Zhang

**Affiliations:** 1 College of Physical Education and Health, Anhui University of Chinese Medicine, Hefei, China; 2 College of Sports Medicine, Wuhan Sports University, Wuhan, China; 3 Institute of Acupuncture and Meridians, Anhui University of Chinese Medicine, Hefei, China

**Keywords:** betaine, exercise mimetics, obesity, diabetes, liver disease, cardiovascular diseases, kidney disease, Alzheimer’s disease

## Abstract

Betaine, a natural compound found in beets, wheat germ, shellfish, and mammalian tissues, plays a crucial role in preventing and treating various chronic diseases. As the global population ages, chronic diseases are posing the primary threat to the health of the elderly, significantly increasing the medical pressure on families and society. Chronic diseases associated with aging involve complex molecular mechanisms and, therefore, developing multipronged interventions is crucial for their prevention and treatment. Although exercise is a primary intervention for preventing and treating chronic diseases, many elderly individuals have motor disabilities. Therefore, researchers are exploring natural products that mimic the therapeutic effects of exercise in individuals who are unable to exercise. Betaine has exhibited significant preventive and therapeutic effects in studies on chronic diseases and is known as an exercise mimetic. A deeper understanding of betaine may help elucidate crucial molecular mechanisms underlying its effects and offer theoretical insights for developing exercise-mimicking foods, supplements, and drugs, which are expected to benefit the human health.

## 1 Introduction

In 2021, noncommunicable diseases resulted in 1.73 billion disability-adjusted life years (1). Chronic diseases are becoming increasingly common in younger populations. According to the 2025 World Health Statistics, 18 million people aged <70 years died of noncommunicable diseases globally in 2021, accounting for more than half of all deaths in this age group (2). Among Chinese people aged >70 years, the prevalence of multiple chronic diseases is 40% (3). Therefore, prevention and treatment of chronic diseases have become major global public health issues. Although clinicians and researchers have been successful in mitigating the progression of chronic diseases to a certain extent through surgery and drugs, the interventions are associated with high medical costs, reduced quality of life, and increased risk of complications (4). Early prevention and treatment may reduce disease incidence, with exercise playing a crucial role (5). The World Health Organization (6) and American College of Sports Medicine (7) recommend that older adults engage in physical activity at least 2–3 times per week to maintain cardiorespiratory, musculoskeletal, and neuromotor fitness. However, effective exercise is not feasible for individuals who are elderly, bedridden, or suffering from motor function loss. Therefore, researchers in sports medicine are exploring drugs, natural products, and synthetic small molecules that can replace exercise. Compared with drugs, small-molecule agonists, and/or inhibitors, natural ingredients may be safer and more suitable for long-term use and serve as sources of motion mimetics. Betaine, a trimethylglycine abundant in plants, is a primary osmoprotectant and methyl donor that plays a significant role in preventing and treating chronic diseases. It has also been identified as an exercise mimetic. This article summarizes the role of betaine in the prevention and treatment of chronic diseases, providing molecular insights and a theoretical basis for developing functional foods, supplements, and drugs.

## 2 Betaine

**Figure 1:**
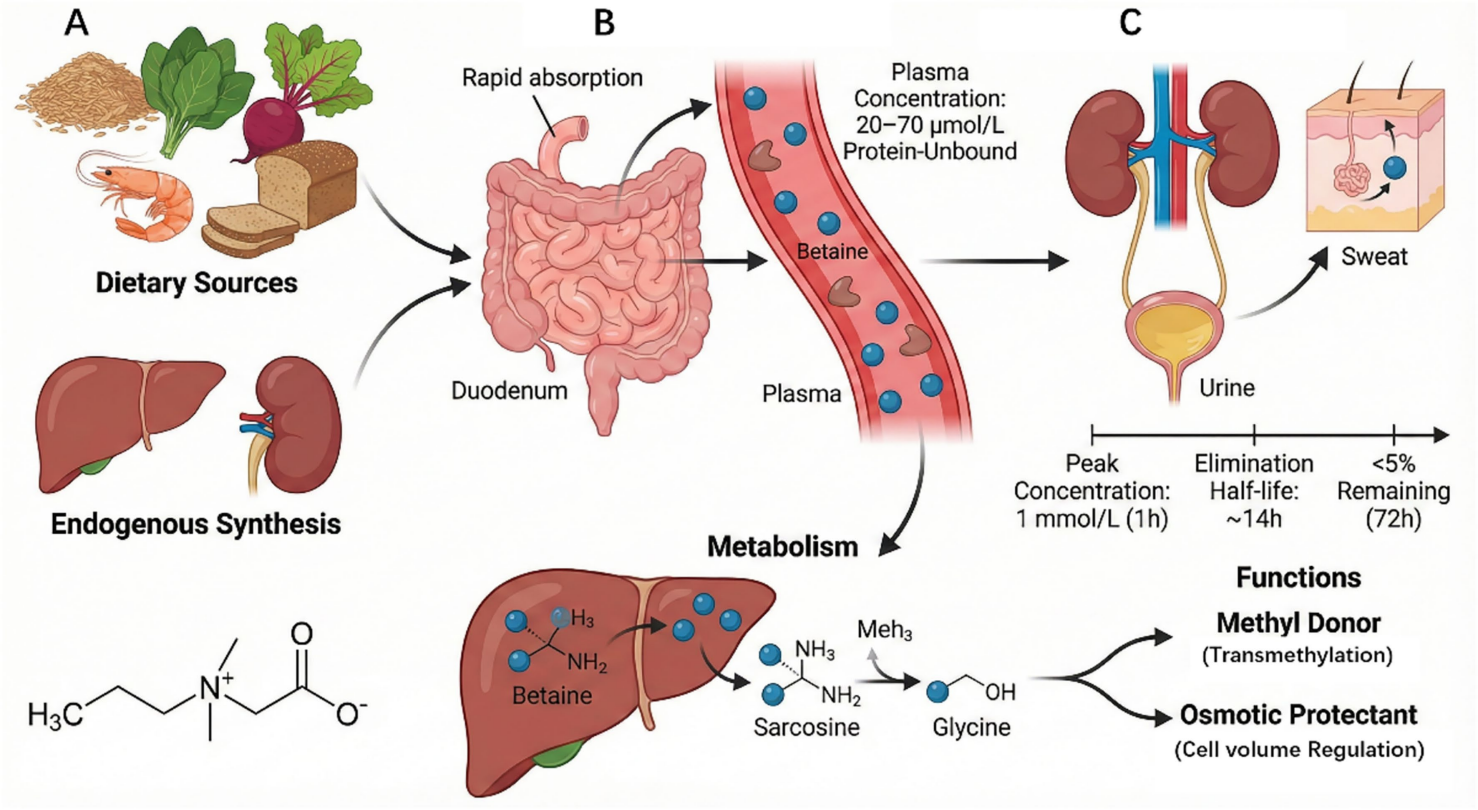
Schematic depicting the sources, synthesis, and kinetics of betaine. Betaine entering the circulatory system is derived from the consumed food and via synthesis in the liver **(A)**; it is converted by the liver into methyl donors, such as S-sarcosine and glycine **(B)**, as well as osmoprotectants, which participate in transmethylation and cell volume regulation, respectively. Some amount of betaine and its metabolites is excreted from the body through urine and sweat **(C)**.

Betaine (C_5_H_11_NO_2_) is a trimethyl derivative of glycine, with a molecular weight of 117.146 g/mol. This stable, nontoxic natural substance, discovered as a byproduct of beet processing, is widely present in microorganisms, plants, and animals. Dietary sources include wheat bran (1,339 mg/100 g), spinach (600–645 mg/100 g), beets (114–297 mg/100 g), shrimp (219 mg/100 g), and whole wheat bread (20 mg/100 g). It is also synthesized by the kidneys and liver (8, 9) (Figure 1A). The median daily betaine intake for the general population is 224.77 mg (10), typically ranging between 100 and 300 mg (11). It is rapidly absorbed through the duodenum, remains unbound to proteins, with its plasma content being 20–70 μmol/L (9, 12) (Figure 1B). Because betaine can pass freely through the kidneys in its unmetabolized form, it is primarily converted into methionine, S-adenosylmethionine (SAM), and dimethylglycine in the body, with only small amounts excreted through urine and sweat. A single dose of betaine (50 mg/kg) in healthy young men reached a peak concentration of 1 mmol/L within 1 h. The elimination half-life of a single dose is approximately 14 h, and <5% of the dose remains after 72 h (13) (Figure 1C). Biologically, betaine functions primarily as a methyl donor in transmethylation and as an osmoprotectant. It participates in various biological processes by gradually removing methyl groups and forming sarcosine and glycine through decomposition and metabolism. As an osmoprotectant, betaine primarily protects cells from osmotic/ionic stress by regulating the concentration and volume of intracellular fluid. Additionally, it exhibits positive regulatory effects in various chronic disease models (14, 15). Betaine regulates cell signals, playing a significant role in disease prevention and treatment.

## 3 Betaine metabolism

**Figure 2:**
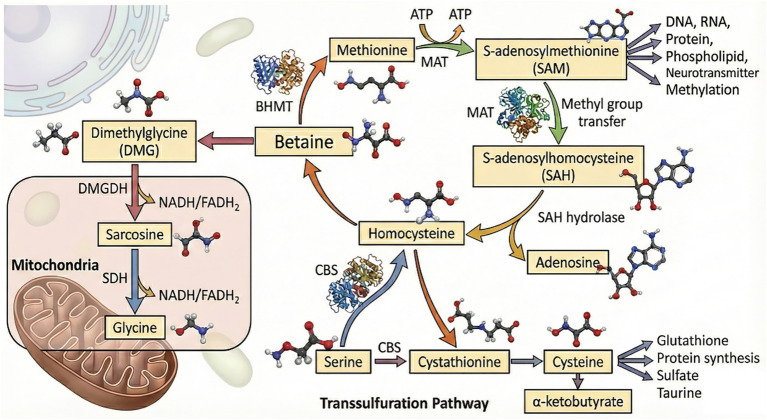
Metabolism of betaine. In a reaction catalyzed by betaine-homocysteine methyltransferase (BHMT), betaine loses a methyl group to form dimethylglycine, which is subsequently demethylated by dimethylglycine dehydrogenase (DMGDH) to form sarcosine that enters the electron transport chain. Sarcosine dehydrogenase (SDH) removes the last methyl group of sarcosine to form glycine. Homocysteine obtains the methyl group transferred by BHMT-catalyzed betaine to form methionine that is converted into S-adenosylmethionine (SAM) by methionine adenosyltransferase (MAT) via transfer of adenosyl group from adenosine triphosphate (ATP). SAM provides methyl groups for the methylation of DNA, RNA, proteins, phospholipids (phosphatidylcholine), and neurotransmitters.

Betaine transfers a methyl group to homocysteine to form methionine via a reaction catalyzed by betaine-homocysteine methyltransferase (BHMT). After losing its methyl group, betaine is converted into dimethylglycine, which undergoes oxidation in the mitochondria. Dimethylglycine dehydrogenase (DMGDH) removes a methyl group to produce sarcosine that enters the electron transport chain, generating reduced nicotinamide adenine dinucleotide/reduced flavin adenine dinucleotides (NADH/FADH2). Sarcosine dehydrogenase (SDH) removes the last methyl group of sarcosine to form glycine, generating NADH/FADH2. Methionine is converted to S-adenosylmethionine (SAM) via the transfer of adenosyl group from adenosine triphosphate (ATP) by methionine adenosyltransferase (MAT). SAM is the primary methyl donor for methylation reactions in the body, providing methyl groups for the modification of DNA, RNA, proteins, phospholipids (phosphatidylcholine), and neurotransmitters. After losing its methyl group, SAM is converted to S-adenosyl-l-homocysteine (SAH)—a potent methyltransferase inhibitor. Increased levels of SAH inhibit methylation reactions, making its rapid metabolism crucial. SAH is hydrolyzed by S-adenosylhomocysteine hydrolase, resulting in the formation of homocysteine and adenosine. Homocysteine can reenter the BHMT pathway and get methylated by betaine to methionine, thereby completing the cycle. If the body is deficient in betaine or folic acid and requires cysteine, homocysteine cannot be remethylated to methionine. Instead, it combines with serine to generate cystathionine through cystathionine β-synthase (CBS), which is then cleaved by cystathionine *γ*-lyase to generate cysteine and *α*-ketobutyrate. Cysteine contributes to glutathione and protein synthesis and can be oxidized and decomposed into sulfate for excretion or is converted to taurine (Figure 2). Therefore, the contribution of betaine to the methionine cycle is crucial for maintaining normal cell function, gene expression regulation, antioxidant defense, and numerous biosynthetic pathways.

## 4 Molecular mechanisms underlying the exercise-mimicking effects of betaine

Betaine is involved in a variety of molecular mechanisms in the body. It exhibits similar effects as exercise in delaying aging and promoting health and is, therefore, considered an exercise mimic (16). Exercise is widely advocated as an intervention to promote health and prevent disease; it mainly activates AMPK through energy consumption and is involved in the regulation of multiple network mechanisms (17). Exercise can enhance the antioxidant, anti-inflammatory, and antiapoptotic effects, regulate immunity, activate autophagy, improve the mitochondrial quality, tissue integrity, circadian rhythm, genetics, endocrine system, and gut microbiota, and prevents and delays chronic diseases, such as cognitive decline and skeletal muscle atrophy (18). Betaine can also activate AMPK, reduce oxidative stress, endoplasmic reticulum stress, inflammation, and apoptosis, delay aging and cancer development, and plays a positive regulatory role in maintaining tissue integrity and mitigating chronic diseases (19, 20). Notably, the role of betaine in mimicking exercise is mainly reflected in activating AMPK (20), improving mitochondrial quality (21), regulating autophagy (22), reducing oxidative stress (23), inhibiting inflammation (24), and genetic modification (25). These are also important mechanisms for maintaining health and preventing and treating diseases. These studies have provided insights into the effects of betaine at the molecular level and form a theoretical basis for developing it as a functional food, nutritional supplement, and drug, benefitting human health.

## 5 Betaine and chronic diseases

Research on the use of betaine in the prevention and treatment of various chronic diseases is ongoing. Below, we provide a review of the literature on the effects of betaine in diseases, such as obesity, diabetes, liver disease, cardiovascular disease, kidney disease, and Alzheimer’s disease, and discuss the underlying mechanisms.

### 5.1 Betaine for the prevention and treatment of obesity

With industrialization, physical activities performed by humans have considerably decreased, resulting in energy surplus in the body, which leads to obesity—a global public health concern worldwide associated with various chronic diseases. Betaine has been widely studied for the prevention and treatment of obesity. Epidemiologically, people with a higher intake of betaine in their daily diet generally have a lower body weight (10, 26), which may be a consequence of reduced body fat (27). In middle-aged and elderly Chinese men, serum betaine levels were positively associated with lean body mass in the whole body, trunk, and limbs (28). In women, betaine supplementation did not enhance exercise performance but significantly reduced the percentage and mass of body fat (29). These results indicate that the effect of betaine in preventing obesity may not be affected by factors, such as age, total calorie intake, physical activity level, and trunk fat, consistent with the results of relevant studies on Newfoundland residents (30). However, supplementation with exogenous betaine had no significant effect on body weight, composition, or resting energy expenditure (31), indicating that a larger sample size and detailed studies are required for confirmation. However, betaine resistance may exist in obesity and prediabetes, possibly associated with low DMGDH levels that limits betaine metabolism (32). Therefore, the effects of betaine on weight management may require larger sample sizes and detailed studies.

**Figure 3:**
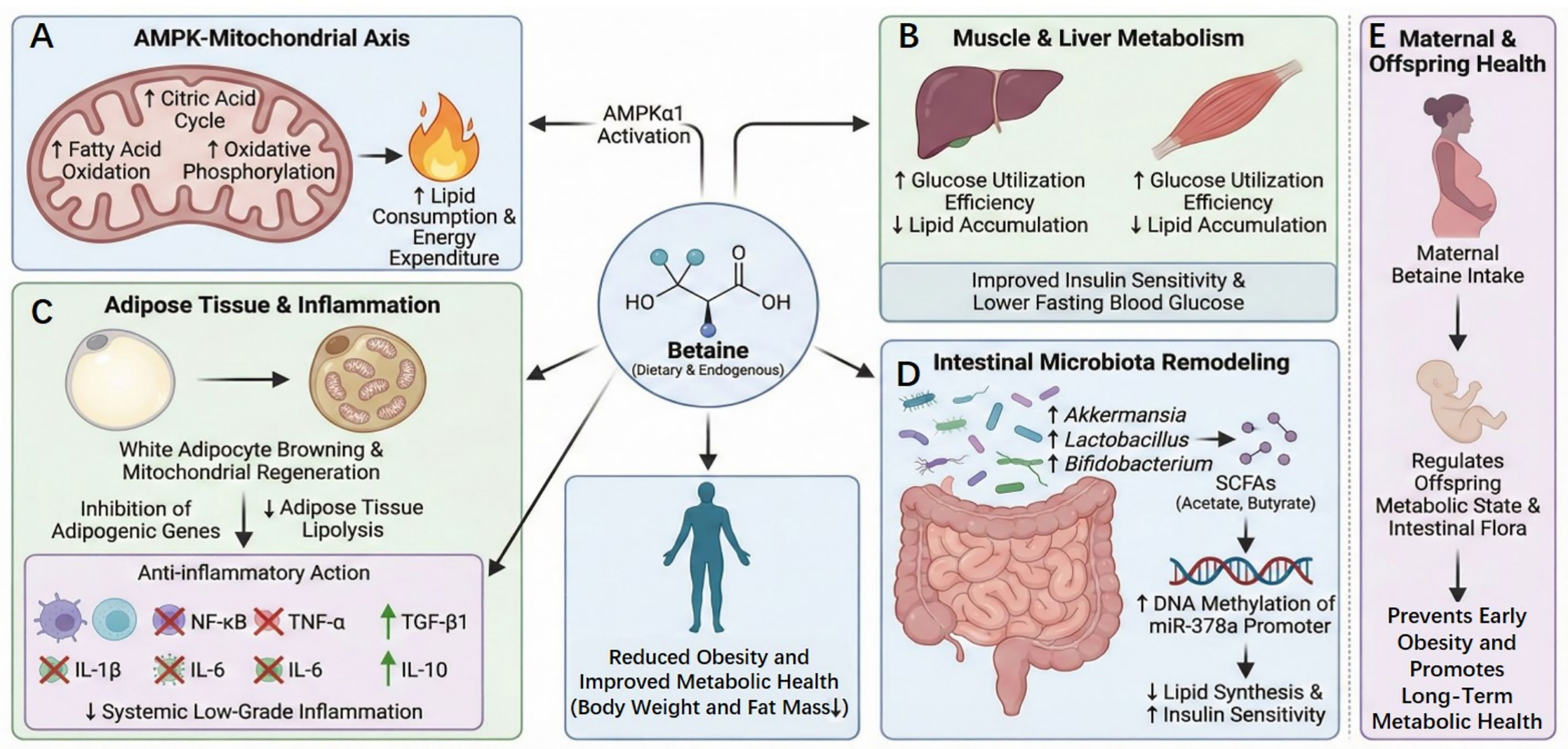
Molecular mechanisms underlying the effects of betaine in preventing and enhancing obesity. Betaine increases energy expenditure by activating the adenosine monophosphate-activated protein kinase–mitochondrial axis, thereby enhancing mitochondrial oxidative phosphorylation **(A)**; improving hepatic and skeletal muscle glucose utilization, reducing lipomatosis, and improving insulin sensitivity and fasting blood glucose **(B)**; promoting white adipose tissue consumption and mitochondrial regeneration, and inhibiting adipogenesis genes, fat deposition, and inflammation **(C)**; increasing DNA methylation through the gut microbiota-short-chain fatty acid-epigenetic cascade, thereby inhibiting lipid synthesis and enhancing insulin sensitivity **(D)**; and regulating metabolic homeostasis and gut microbiota in the offspring to prevent early-onset obesity and improve metabolic health **(E)**.

The beneficial effects of betaine on obesity and related metabolic disorders have been confirmed in numerous studies. These effects involve various complementary pathways, such as energy metabolism, inflammation regulation, and gut microecological remodeling. *In vivo* experiments have demonstrated that betaine activates adenosine monophosphate-activated protein kinase subunit alpha 1 (AMPKα1), upregulating fatty acid oxidation, citric acid cycle, and mitochondrial oxidative phosphorylation, thereby accelerating lipid consumption and limiting weight gain (33, 34) (Figure 3A). In a high-fat, high-sugar diet-induced obese mouse model, betaine intervention significantly enhanced glucose utilization efficiency in the skeletal muscle and liver, lowered fasting blood glucose levels, and alleviated systemic inflammation (35) (Figure 3B). If combined with exercise training, the blood sugar-lowering effect is more significant (36). Betaine facilitates mitochondrial regeneration in white adipose tissue, induces browning of white adipocytes, and inhibits the expression of adipogenic genes. Additionally, it reduces the mRNA levels of proinflammatory cytokines, such as interleukin-1 (IL-1), interleukin-6 (IL-6), and interleukin-12 (IL-12), both in muscle tissue *in vivo* and in adipose tissue cells *in vitro*, thereby alleviating high-fat diet-induced obesity and insulin resistance (37, 38), consistent with the results of facilitating the reduction of skeletal muscle lipid deposition and enhancing adipose tissue lipolysis (39) (Figure 3C). *In vitro* studies have confirmed that betaine can inhibit the expression of hypoxia-induced IL-6 and tumor necrosis factor-alpha (TNF-*α*) mRNAs in human adipocytes, indicating that it directly acts on adipose tissue to reduce obesity-related low-grade inflammation (38). Moreover, it exhibited conservative metabolic-protective effects in lower vertebrate models. After betaine treatment of high-fat-induced obese black sea bream juveniles, the silent information regulator transcript 1/sterol regulatory element-binding protein 1/peroxisome proliferator-activated receptor alpha (SIRT1/SREBP-1/PPAR*α*) axis was activated, anti-inflammatory cytokines transforming growth factor-beta 1 (TGF-β1) and IL-6 were upregulated, whereas proinflammatory signals, such as nuclear factor kappa-light-chain-enhancer of activated b cells (NF-κB), TNF-α, and interleukin-1 beta (IL-1β) were downregulated, confirming its cross-species anti-inflammatory activity (40). However, the conclusions of these population-based studies are inconsistent. Certain randomized controlled trials have demonstrated that betaine reduces the levels of circulating inflammatory markers; however, the differences were not statistically significant. This may be associated with the small sample size, short intervention period, and numerous confounding factors. A large sample size and long-term intervention are required for validation (41). Additionally, betaine exerts antiobesity effects by reshaping the gut microbiota and its metabolites. In a high-fat diet mouse model, betaine facilitated the proliferation of beneficial bacteria, such as *Akkermansia muciniphila*, *Lactobacillus*, and *Bifidobacterium*, and increased the production of acetate and butyrate. Short-chain fatty acids (SCFAs) inhibit lipid synthesis and enhance insulin sensitivity by increasing the DNA methylation of the miR-378a promoter (42, 43) (Figure 3D). A dose-effect study further confirmed that betaine intervention at a dose of 120 mg/kg body weight for 4 weeks was adequate to significantly enhance glycemic and lipid profiles and reduce body weight in mice fed a high-fat diet (44).

The role of betaine is also reflected in maternal and child health. Maternal betaine intake affects obesity risk in the offspring. In experiments on obese rats, betaine supplementation during pregnancy helped the offspring restore normal growth and developmental rhythms while reducing obesity (45). Whether the mother consumes betaine through diet during pregnancy or adds it to breast milk, it can regulate the metabolic state and gut microbiota composition of the offspring, thereby preventing early obesity and facilitating long-term metabolic health (46, 47) (Figure 3E). Additionally, human studies have confirmed that betaine content in the maternal body is associated with the birth weight and abdominal fat mass in babies. In particular, higher maternal betaine levels are associated with healthier birth weight and lower abdominal fat mass in infants (48).

Betaine synergistically enhances obesity and its metabolic complications through various targets via the AMPK-mitochondrial axis, SIRT1/SREBP-1/PPARα signaling, inhibition of inflammation, and the microbiota-SCFA-epigenetic cascade, providing a mechanistic basis for its clinical translation.

### 5.2 Betaine for the prevention and treatment of diabetes

Diabetes is a common metabolic disease, the onset of which is primarily caused by the combined effects of genetic susceptibility and acquired environmental factors (long-term high-sugar and high-fat diets, sedentary lifestyles, and obesity). The core pathological mechanism is insulin resistance in peripheral tissues and organs (reduced insulin sensitivity of muscles, fat, and liver) and reduced pancreatic β-cell function (insufficient or delayed insulin secretion), resulting in increased blood levels (49). No obvious symptoms might be present in the early stages of the disease; however, as the disease progresses, typical manifestations, such as polydipsia, polyphagia, polyuria, and weight loss occur. If blood sugar is not well controlled for a long time, it gradually damage tissues and organs throughout the body and may induce chronic complications, such as cardiovascular disease, kidney disease, neuropathy, and retinopathy (50).

#### 5.2.1 Type 2 diabetes mellitus

**Figure 4:**
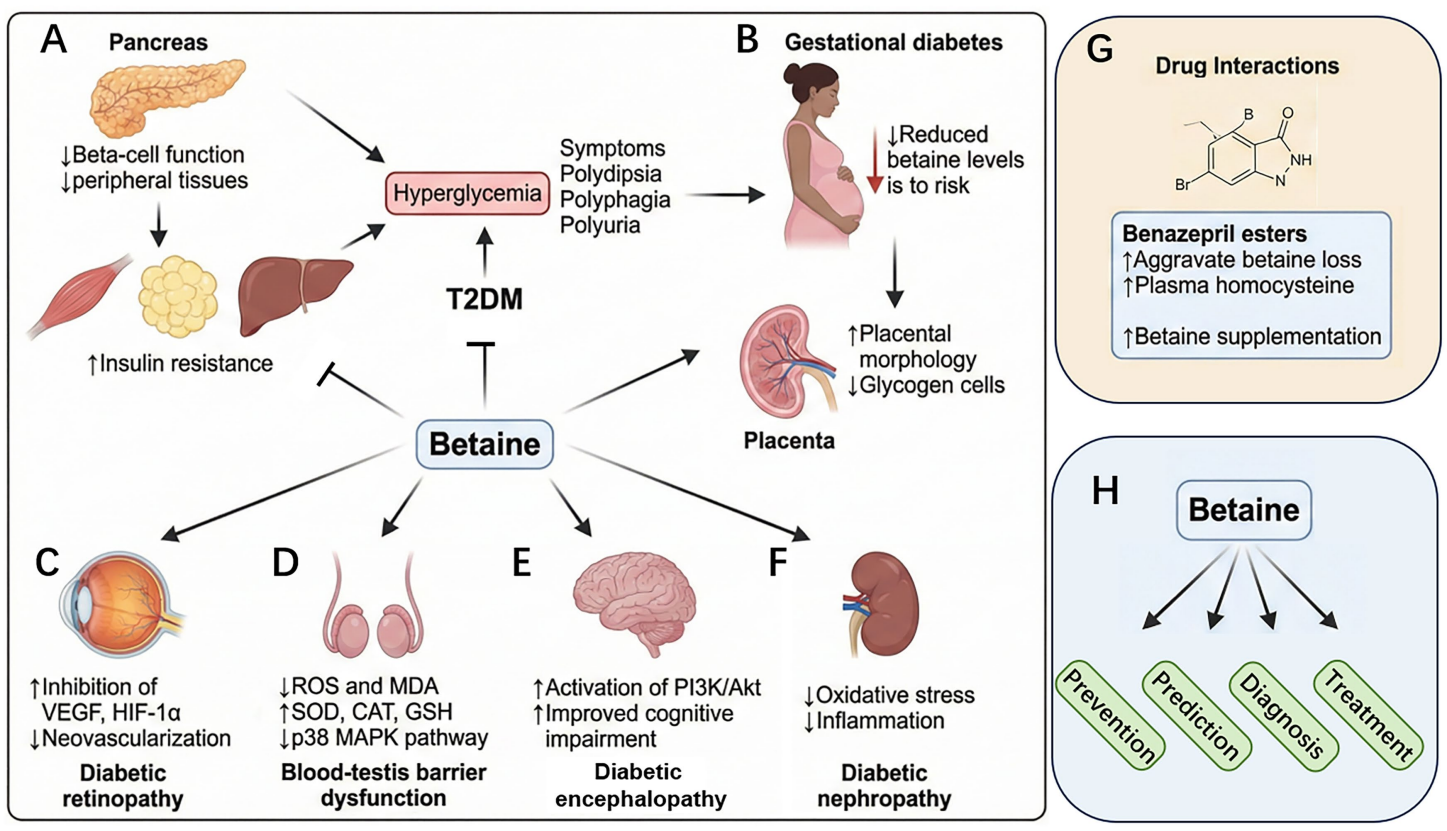
Molecular mechanisms underlying the effects of betaine in regulating certain tissues and functions in type 2 diabetes mellitus (T2DM), gestational diabetes, and diabetic complications. **(A)** In T2DM, betaine lowers blood lipids, HbA1c homeostatic model assessment of insulin resistance, serum glucose, and insulin resistance. **(B)** Betaine reduces the risk of gestational diabetes, mitigates DNA damage in placental and embryonic tissues, and increases the thickness of the placental attachment zone and labyrinth. **(C)** Betaine reduces retinal Akt/vascular endothelial growth factor/hypoxia-inducible factor 1-alpha and alleviates retinopathy. **(D)** In the testicular tissue, betaine reduces superoxide dismutase (SOD), malondialdehyde (MDA), and p38 mitogen-activated protein kinase signaling pathways and increases superoxide dismutase (SOD), catalase, and glutathione levels. **(E)** In the brain tissue, betaine activates phosphatidylinositol 3-kinase/protein kinase B (Akt) and enhances cognitive ability. **(F)** In the renal tissue, betaine reduces reactive oxygen species (ROS) and malondialdehyde (MDA) levels. **(G)** Benazepril and its analogs appear to exacerbate betaine loss, leading to elevated plasma homocysteine levels, suggesting that betaine supplementation should be considered. **(H)** Betaine plays preventive, predictive, diagnostic, and therapeutic roles in diabetes-related diseases.

The baseline betaine level in patients with type 2 diabetes mellitus (T2DM) is lower than that in healthy individuals (51); however, hyperglycemia is not the cause of increased betaine excretion (52). Nevertheless, it is significantly correlated with glycated hemoglobin levels (53). This is consistent with the results of another study that used liquid chromatography-tandem mass spectrometry to measure betaine in urine and plasma and a multivariate regression analysis to show that glycated hemoglobin was the strongest determinant of betaine excretion in patients with diabetes (54), although different test results have also been reported (55). Betaine is a marker of diabetes risk in high-risk individuals and has been reported to perform a direct role in regulating metabolic health (56). Lower betaine intake is associated with lower insulin levels, homeostatic model assessment of insulin resistance (HOMA-IR) (57), and an increased risk of other T2DM (58). For example, plasma betaine content in patients with T2DM is negatively associated with the occurrence of microvascular complications (59). In a population-based study in Newfoundland, high serum betaine levels were associated with low lipid levels and insulin resistance (60) (Figure 4A). In adults, higher betaine levels were negatively associated with lower diabetes-related markers, serum insulin concentrations, and HOMA-IR (57). However, no significant association between betaine use and diabetes was observed in some other studies. A follow-up study involving 13,440 participants demonstrated that dietary betaine intake was not associated with T2DM (61). A semi-quantitative food frequency questionnaire survey involving 6,022 participants aged ≥18 years in Tehran did not find any association between dietary betaine intake and T2DM (62). These two studies involved a large number of participants. Because of the numerous factors affecting human health, semi-quantitative food frequency questionnaires may not accurately reflect this phenomenon. Therefore, more rigorous clinical and basic studies are required to verify the relationship between betaine and T2DM.

#### 5.2.2 Gestational diabetes

Gestational diabetes is a glucose metabolism disorder that often occurs during pregnancy. Reduced serum betaine levels are an independent risk factor for gestational diabetes that may be associated with blood sugar regulation and short-term fluctuations (63). Higher dietary betaine intake among pregnant women in China was reported to be inversely associated with the risk of gestational diabetes mellitus in women without a history of childbearing (64). Betaine administration to mice after feeding a high-fat diet for 4 weeks before and during pregnancy to induce gestational diabetes alleviated morphological alterations in the placental junction area and increased the glycogen cell area in pregnant mice. Additionally, *in vitro* experiments have demonstrated that betaine can alter certain determinants of placental transport during the hyperglycemic response (65). In a streptozotocin (STZ)-induced gestational diabetes model, betaine significantly increased insulin levels, restored normal plasma total homocysteine concentrations, and enhanced insulin resistance and blood lipid status (66). Additionally, betaine could reduce increased DNA damage levels in the placental and embryonic tissues of rats at 14–20 days of gestation, with the best effect achieved when betaine was administered orally at a dose of 100 mg/kg body weight (67) (Figure 4B).

#### 5.2.3 Diabetic complications

Basic research on the effects of betaine on diabetes is increasing, with some studies demonstrating positive results. Betaine can inhibit the activation of protein kinase B (Akt) in the retina of STZ-induced diabetic rats, thereby attenuating the increase in vascular endothelial growth factor (VEGF) and hypoxia-inducible factor 1-alpha (HIF-1α) expression and inhibiting neovascularization to delay diabetic retinopathy-associated complications (59) (Figure 4C). In the STZ-induced diabetic mouse model, oral administration of betaine reduced the levels of reactive oxygen species (ROS) and malondialdehyde (MDA) in the testicular tissue and increased the activities of superoxide dismutase (SOD), catalase (CAT), and glutathione (GSH), thereby inhibiting the p38 mitogen-activated protein kinase (p38 MAPK) signaling pathway and protecting against blood-testis barrier dysfunction (68) (Figure 4D). Similarly, in STZ-induced diabetic rats, betaine inhibited oxidative stress and inflammation while activating the phosphatidylinositol 3-kinase (PI3K)/Akt signaling pathway to enhance cognitive impairment (69) (Figure 4E). In a diabetic *db/db* mouse model, betaine alleviated endoplasmic reticulum and oxidative stress to enhance insulin resistance, hyperlipidemia, and tau protein hyperphosphorylation (70). In STZ-induced male diabetic rats, betaine reduced the increase in the levels of glycosylated hemoglobin in in blood, serum glucose, and lipids, and the pro-oxidative state in the liver and kidney (71) (Figure 4F). Betaine exerts a preventive effect, even in cases of diabetes caused by arsenic poisoning-induced impaired glucose tolerance (72). However, the use of certain drugs may result in the abnormal excretion of betaine from the body. Benazepril esters appear to aggravate betaine loss, resulting in increased plasma homocysteine levels (Figure 4G). Therefore, betaine supplementation should be considered when treating patients with fibric acid drugs (73).

Studies in human subjects and basic experiments show that betaine may have significant potential in the prevention, prediction, diagnosis, and treatment of diabetes and diabetic complications (Figure 4H). Although certain results were inconsistent, more authoritative experiments are required for verification of these findings.

### 5.3 Betaine for the prevention and treatment of liver disease

As a highly complex chemical factory, the liver plays a crucial role in metabolism and processing of proteins, fats, sugars, vitamins, and hormones. With modern lifestyle, liver disease has become common worldwide. Metabolic fatty liver disease affects 30% of the global population (74); however, no treatment is available for alcoholic liver disease (75). The effects of betaine on the liver have been assessed for decades, and positive effects via various molecular mechanisms have been reported.

#### 5.3.1 Metabolic fatty liver

Metabolic fatty liver disease often coexists with metabolic disorders, such as obesity and metabolic syndrome, and can result in reduced betaine levels in the body (76). Betaine primarily regulates hepatic lipid metabolism by inhibiting lipogenesis and facilitating fatty acid oxidation. In the classic *db/db* mouse model of metabolic fatty liver, oral administration of betaine can inhibit the interaction between forkhead box protein O6 and peroxisome proliferator-activated receptor gamma (PPARγ), inhibit the expression of primary lipogenic genes, such as fatty acid synthase (FAS) and acetyl-CoA carboxylase, and reduce hepatic lipid accumulation (77, 78). Simultaneously, betaine can significantly increase the expression of genes (carnitine palmitoyltransferase 1 and PPARα) and enhance fatty acid oxidation and lipid transport, thereby alleviating fat accumulation in the liver (79, 80). In a vitamin B6 deficiency-induced hepatic fat deposition model, betaine exhibited a significant inhibitory effect and restored methionine metabolism and very low-density lipoprotein secretion, the mechanisms of which may be associated with the restoration of the phosphatidylethanolamine-to-phosphatidylcholine conversion pathway (81, 82). Epigenetic regulation is another significant regulatory mechanism. For example, in a hen model, betaine inhibited lipogenesis and facilitated lipid decomposition by altering DNA methylation levels or mRNA N6-methyladenosine (m^6^A) modification in the promoter region of genes, such as sterol regulatory element-binding protein 1, FAS, and stearoyl-CoA desaturase, thereby reducing hepatic triglyceride deposition (83). The addition of demethylase blocked the regulatory effects of betaine on lipid metabolism and mitochondrial content, further confirming that betaine affects RNA methylation (84). Regarding glucose metabolism and whole-body energy balance, betaine can enhance the activity of enzymes associated with glucose uptake, glycogen synthesis, and decomposition in the liver and muscles, indicating its potential to regulate glucose metabolism disorders (35). Additionally, betaine supplementation can increase the levels of fibroblast growth factor 21 (FGF21) in the liver and circulation, enhance white fat oxidation capacity and whole-body energy expenditure, thereby enhancing blood glucose homeostasis and metabolic health (79, 83, 85). Moreover, betaine exhibits antioxidant, anti-inflammatory, and gut microbiota-regulatory functions. In a high fructose-induced rat model, betaine reversed the increased levels of oxidative stress indicators and inflammatory factors and alleviated liver damage (86). In fish, betaine enhanced the structure of the gut microbiota, regulated trimethylamine metabolism and bile acid metabolism, and indirectly alleviated liver fat accumulation induced by a high-carbohydrate diet (87). Notably, the protective effect of betaine is consistent across species and has been verified in various models, such as mice, rats, fish, and poultry, even demonstrating a preventive effect during the embryonic or maternal supplementation stages (80, 83, 88). However, certain studies have indicated that although it facilitates growth in a growing pig model, it does not significantly affect fatty acid oxidation, indicating that the mechanism may be species-specific (89). Betaine regulates liver and systemic metabolism through various pathways and demonstrates broad potential for preventing and alleviating metabolic fatty liver disease. However, its specific mechanism of action varies based on the model and species, and further research is required to clarify this mechanism.

#### 5.3.2 Nonalcoholic fatty liver disease

**Figure 5:**
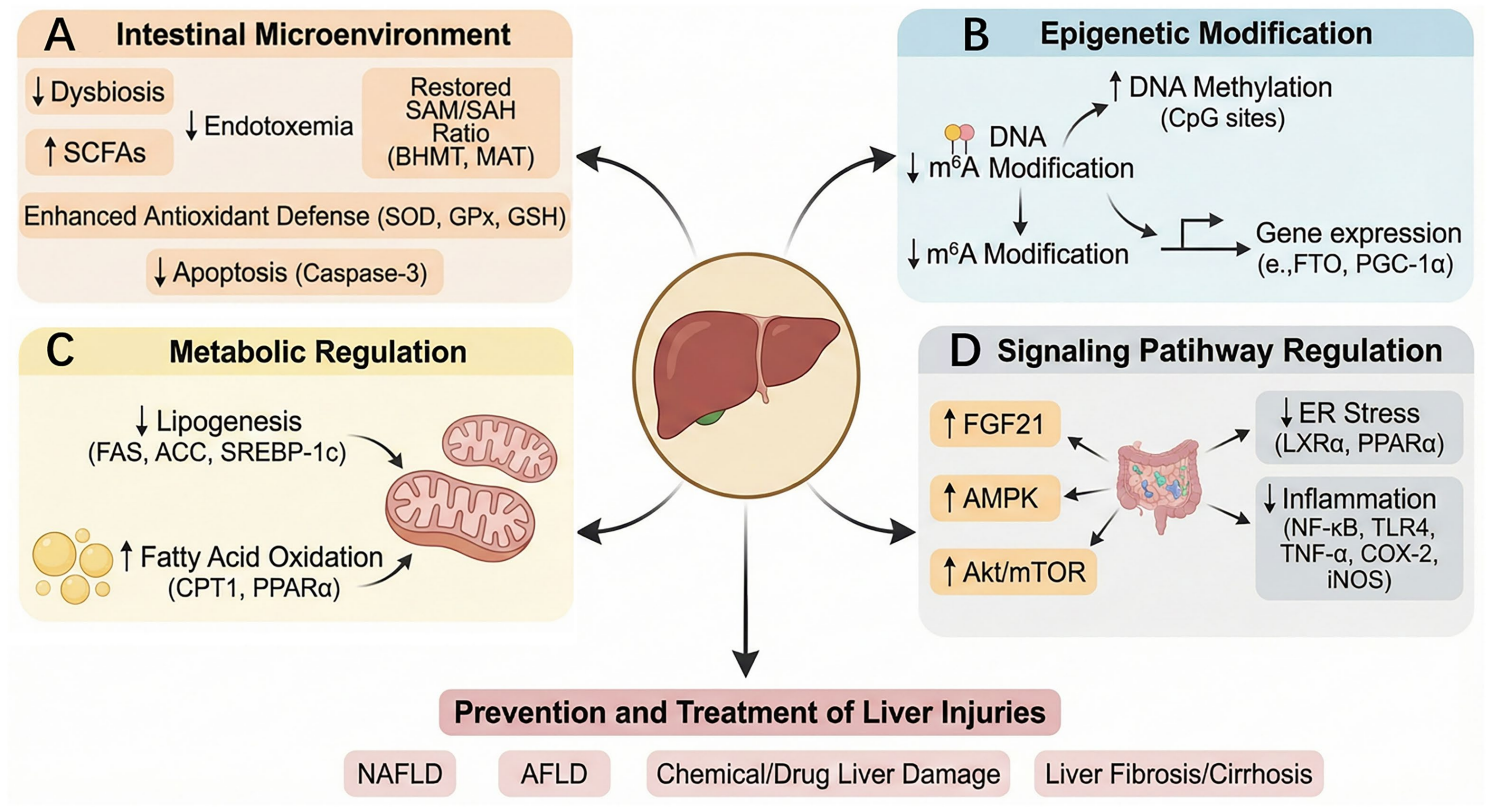
Mechanisms underlying the protective effects of betaine in various liver diseases and injury. Betaine works by improving the gut dysbiosis, increasing short-chain fatty acid levels, reshaping the SAM/SAH ratio, enhancing antioxidant defense, and inhibiting apoptosis **(A)**; regulating the expression of genes such as PGC-1α through m^6^A methylation of DNA **(B)**; downregulating the levels of genes and proteins related to lipogenesis and upregulating the levels of genes and proteins related to fatty acid oxidation (FAS, ACC, SREBP-1c↓; CPT-1PPAR*α*↑) **(C)**; and participating in the regulation of molecular signaling pathways related to AMPK, Akt/mTOR, endoplasmic reticulum stress, and inflammation **(D)**, thereby preventing and treating liver damage caused by NAFLD, AFLD, liver fibrosis/cirrhosis, and chemical/drug liver damage.

Betaine exhibits multilevel protective effects against nonalcoholic fatty liver disease (NAFLD) through mechanisms involving metabolic regulation, epigenetic modification, signaling pathway regulation, and enhancement of the gut microenvironment. Among these, epigenetic regulation plays a crucial role. As a methyl donor, betaine increases overall methylation levels and regulates abnormal DNA methylation [by regulating cytosine-phosphate-guanine (CpG) methylation in the promoter region of PPARγ and hepcidin antimicrobial peptide genes associated with lipogenesis and iron metabolism]. Additionally, it inhibits the m^6^A hypomethylation state, thereby regulating gene expression to reduce fatty acid synthesis and increase fat decomposition, which reduces lipid accumulation in the liver and protects it (90–93). Betaine can upregulate BHMT, enhance the production of nicotinamide adenine dinucleotide phosphate, and increase the expression of fat and obesity-related protein (FTO), which reduces m^6^A levels in the coding sequence region of the peroxisome proliferator-activated receptor gamma coactivator 1-alpha (PGC-1α) transcript. This upregulates PGC-1α and inhibits lipid accumulation in the liver through the BHMT/FTO/m^6^A/PGC-1*α* pathway to alleviate NAFLD (94) (Figure 5A). Betaine activates various primary signaling pathways involved in metabolic regulation. It can inhibit the expression of lipid metabolism-related genes through the FGF10/AMPK pathway, facilitate fatty acid β-oxidation, and alleviate endoplasmic reticulum stress by restoring the expression of liver X receptor alpha and PPARα. Additionally, it enhances the Akt/mechanistic target of rapamycin signaling pathway, activates autophagy, and stimulates insulin receptor substrate 1 to activate downstream signaling pathways, thereby alleviating hepatic steatosis, gluconeogenesis, and inflammatory response (22, 95–97). Betaine exhibits significant antioxidant and anti-inflammatory effects. It enhances the metabolism of sulfur-containing amino acids to enhance antioxidant defense, and reduce oxidative stress, expression of inflammatory factors [TNF-α, cyclooxygenase-2 (COX-2), and inducible nitric oxide synthase (iNOS)], and cell apoptosis through signaling pathways, such as high mobility group box 1/toll-like receptor 4 (TLR4), thereby restoring liver function (98–100). Additionally, betaine enhances mitochondrial function, reduces the number of swollen mitochondria, and increases autophagosome formation, thereby alleviating damage to liver cells (101). Notably, the effects of betaine were cross-organ and cross-generational. Maternal betaine supplementation can enhance high-fat diet-induced NAFLD by regulating the gut microbiota and SCFAs in the offspring (102) (Figure 5B). Moreover, the protective effects of betaine on the liver may involve intergenerational communication between the liver and brain, such as regulating brain phospholipid metabolism (103). Clinical and pathological evidence supports its effectiveness. Patients with NAFLD often have low betaine levels (104), and clinical interventions have demonstrated that oral betaine can significantly reduce serum alanine aminotransferase and aspartate aminotransferase levels and enhance fat degeneration, inflammatory necrosis, and fibrosis (105). Although betaine failed to enhance hepatic steatosis, it prevented the worsening of hepatic steatosis (106). In animals, betaine can effectively reduce the liver triglyceride content, inhibit liver cell swelling and necrosis, and reduce lipid deposition (107, 108). In summary, betaine exhibits significant potential for preventing and alleviating NAFLD through synergistic effects on various targets and pathways.

#### 5.3.3 Alcoholic fatty liver disease

Betaine exerts a significant protective effect against ethanol-induced alcoholic liver damage through various pathways. Its core mechanism focuses on restoring methyl metabolic homeostasis, enhancing lipid metabolism, and antagonizing oxidative stress-induced damage. The main role of betaine is to reshape methyl metabolism balance in the liver. Betaine supplementation can enhance hepatic methionine metabolism and SAM levels in the early stages, facilitate choline-histidine methyltransferase activity, and reduce hepatic triglyceride accumulation (109). Additionally, betaine significantly upregulates the expression of BHMT-1, methionine adenosyltransferase-1, and glycine N-methyltransferase, effectively increasing the levels of SAM in the liver and restoring the normal SAM/SAH metabolic ratio (110–114). Normalization of this crucial ratio enhances the activity of phosphoethanolamine methyltransferase, restores normal synthesis of phosphatidylcholine to reduce fat deposition (112), reactivates the repair response mediated by protein L-isoaspartate methyltransferase (111), and attenuates liver damage caused by reduced methylation of the protein phosphatase 2a (PP2A) catalytic subunit (115). Notably, betaine can inhibit alcohol-induced increases in hepatocyte SAH (a potent inhibitor of methylation reactions), increase caspase-3 levels, and reduce DNA content, demonstrating therapeutic potential (116). In regulating lipid metabolism, betaine can directly inhibit primary lipogenesis genes, including diacylglycerol O-acyltransferase 1/2, SREBP-1c, and FAS, while upregulating factors, such as PGC-1α, thereby synergistically enhancing alcohol-induced liver lipid accumulation (117, 118) (Figure 5C). Additionally, betaine exhibits strong antioxidant and anti-inflammatory capabilities, alleviating oxidative damage by reversing GSH depletion, increasing MDA levels, reducing vitamin A content, and inhibiting cytochrome p450 family 2 subfamily E member 1 expression and hydroxytaurine production (113, 119). Simultaneously, betaine can inhibit primary inflammatory signaling pathways, such as TLR4, prevent the upregulation of related proinflammatory factors and signaling molecules (TLR2/4, IL-1β, and signal transducer and activator of transcription 3), and alleviate the inflammatory response (120, 121). Notably, betaine may facilitate adrenaline synthesis by affecting phenylethanolamine N-methyltransferase, thereby indirectly increasing the alcohol metabolism rate and preventing excessive alcohol concentrations (122). The study demonstrated that betaine protects the liver in a dose-dependent manner, with 0.5% betaine in feed sufficient to raise SAM levels that prevent fatty liver disease (114). However, the effects of betaine are selective. For example, in certain models, it did not reverse carbonic anhydrase-III downregulation or reduce the expression of TNF-*α* and cluster of differentiation 14 mRNA (110, 123), indicating that its mechanism of action is highly specific. In summary, by correcting the imbalance in methyl metabolism, betaine synergistically regulates lipid metabolism, oxidative stress, and inflammatory pathways, forming a multidimensional protective network against alcoholic liver damage.

#### 5.3.4 Other liver damage types

Betaine protects against various types of liver damage, including chemical poisoning, drugs, liver fibrosis, and cirrhosis. Its efficacy is achieved through various mechanisms, including antioxidant, anti-inflammatory, metabolic, and epigenetic regulation.

##### 5.3.4.1 Chemical and drug-induced liver damage

In liver injury models induced by chemical poisons and drugs, such as carbon tetrachloride, diethylnitrosamine, acetaminophen, thioacetamide, and cisplatin, betaine exerts core protective effects primarily by enhancing antioxidant defense and inhibiting inflammation. Betaine can significantly increase the activity of antioxidant enzymes [SOD and glutathione peroxidase (GPx)], restore GSH levels, reduce MDA content, inhibit the expression of primary inflammatory factors (NF-κB, TNF-*α*, and COX-2), and inhibit lipid deposition and degeneration (124–131) (Figure 5D). Additionally, betaine can protect mitochondrial function by protecting complex II activity and inhibiting apoptosis by inhibiting caspase-3, thereby maintaining hepatocyte structural integrity (21, 126, 131, 132). Betaine may play a protective role against niacin-induced hepatotoxicity by preventing SAM depletion (133).

##### 5.3.4.2 Liver fibrosis and cirrhosis

In a liver fibrosis model induced by a high-fat diet combined with carbon tetrachloride, bile duct ligation, ethanol, and other factors, betaine exerted its effects through the aforementioned antioxidant and anti-inflammatory mechanisms and directly targeted the antifibrosis pathway. Betaine can effectively delay the progression of fibrosis by downregulating TGF-β1, alpha-smooth muscle actin (*α*-SMA), and collagen type I alpha 1 chain (COL1A1), regulating the balance of matrix metalloproteinase-2 and tissue inhibitor of metalloproteinase-1/-2, and inhibiting the activation of hepatic stellate cells (23, 125, 134–136). Clinical observations have demonstrated that plasma betaine levels in patients with cirrhosis are associated with disease severity and decrease after liver transplantation, indicating their association with disease progression (137).

##### 5.3.4.3 Special types of liver injury caused by radiation, ischemia-reperfusion, and viral hepatitis

In radiation-induced liver injury and liver ischemia-reperfusion models, betaine plays a protective role mainly via its strong antioxidant activity, delaying tissue damage (124, 138). In chronic hepatitis C, betaine exhibits immunomodulatory potential and enhances interferon α antiviral signaling by restoring signal transducer and activator of transcription 1 methylation (139).

##### 5.3.4.4 Transgenerational programming effects and developmental regulation

The protective effects of betaine are characteristics of transgenerational epigenetic programming. Maternal betaine intake (rats and pigs) can programmatically alter liver metabolism, cell proliferation, and the developmental trajectory of offspring—and even the F2 generation—by affecting the DNA methylation pattern of the insulin-like growth factor (IGF) gene family (IGF-1/2) in the liver of the offspring and regulating the expression of glucocorticoid receptor signaling and lipolysis genes (adipose triglyceride lipase and hormone-sensitive lipase) (25, 140–145).

Betaine exhibits multitarget protective effects. Its main function is to provide methyl groups and synergistically regulate various pathways—including antioxidative stress, inflammation suppression, metabolic homeostasis, and epigenetic programming—thereby forming a networked protective system. This has the potential for effective prevention and treatment of various liver injuries, including the chemical, fibrotic, and viral types.

### 5.4 Betaine for the prevention and treatment of cardiovascular disease

**Figure 6:**
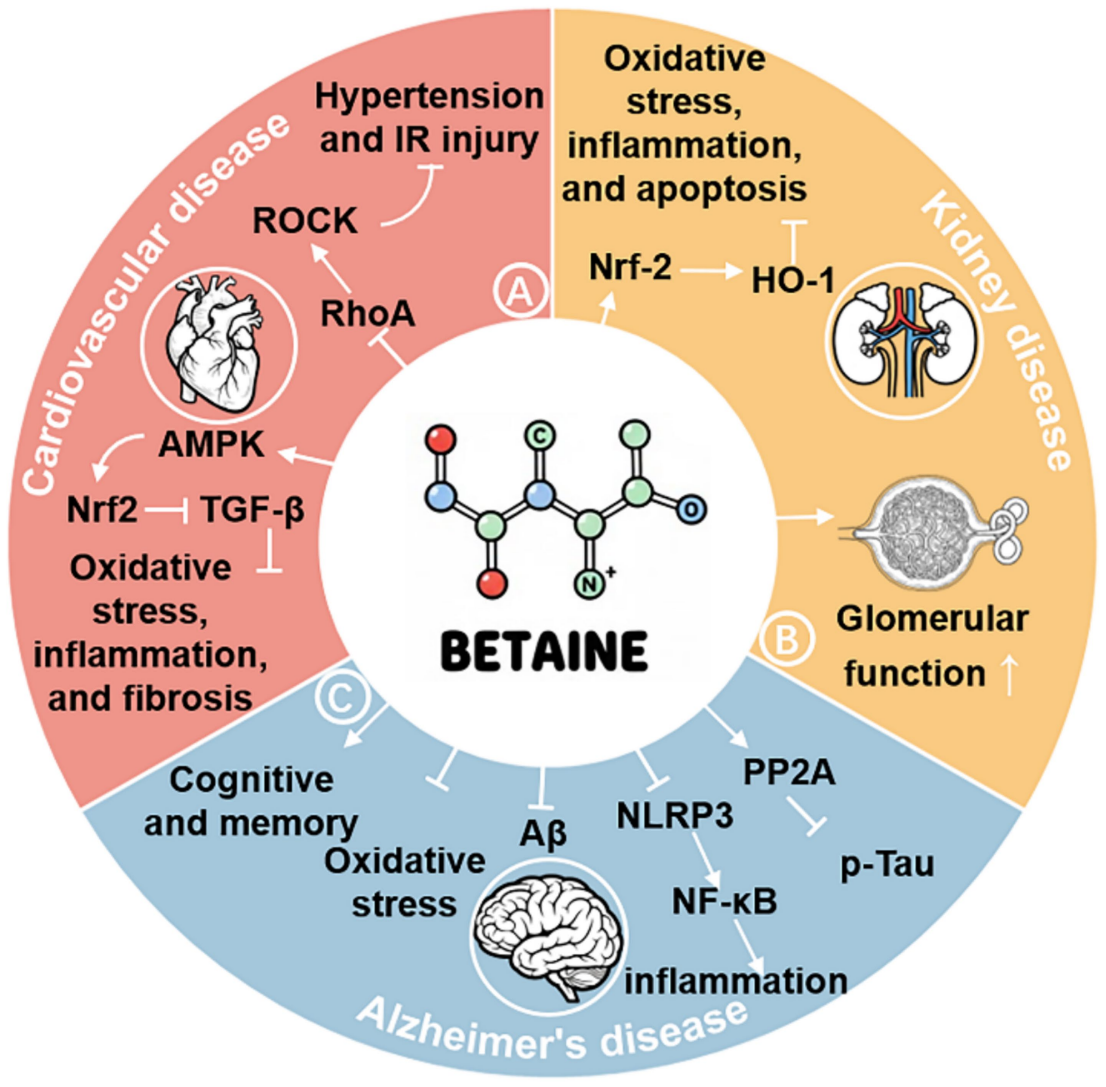
Mechanisms underlying the effects of betaine in preventing and treating cardiovascular, kidney, and Alzheimer’s diseases. Betaine reduces hypertension and ischemia-reperfusion injury by inhibiting Ras homologous family member A/Rho-associated coiled-coil-containing protein kinase and enhances cardiovascular function by activating the adenosine monophosphate-activated protein kinase/nuclear factor erythroid 2-related factor 2 (Nrf2)/transforming growth factor-beta pathway, thereby reducing oxidative stress, inflammation, and fibrosis **(A)**; reduces oxidative stress, neuroinflammation, and apoptosis by activating Nrf2/heme oxygenase-1, thereby enhancing glomerular function and kidney health **(B)**; enhances cognition and memory by inhibiting amyloid-beta production, oxidative stress, NOD-, LRR-, and pyrin domain-containing protein 3/nuclear factor kappa-light-chain-enhancer of activated B cells pathway, and inflammation, and by activating protein phosphatase 2a to inhibit Tau hyperphosphorylation, thereby enhancing the Alzheimer’s disease **(C)**.

Existing studies on the association between betaine and cardiovascular health have yielded inconsistent results. Several large-scale, long-term follow-up studies have demonstrated no significant association between betaine intake and the risk of cardiovascular disease morbidity or mortality in the general adult population (146–149). A study involving 18,076 novel cardiovascular disease events further confirmed the lack of a significant association between the two diseases (148). However, betaine has demonstrated different effects in specific populations or disease contexts. For example, in the Guangdong population of China, higher dietary betaine intake was associated with a lower risk of cardiovascular death (150). In patients with ischemic stroke, betaine helped reduce the risk of recurrent cardiovascular events and enhanced cognitive function (151, 152). In African Americans, dietary betaine intake increased nonlinearly with the risk of coronary heart disease (153). Regarding biomarkers, low-dose betaine supplementation (<4 g/day) can reduce homocysteine levels without causing lipid abnormalities (154), but daily supplementation of ≥4 g may slightly increase total cholesterol levels (155). Betaine levels are inversely associated with blood pressure, particularly in women (156). At the mechanistic level, betaine may exert a cardioprotective effect by inhibiting oxidative stress, inflammation, and fibrosis by regulating the AMPK/nuclear factor erythroid 2-related factor 2 (Nrf2)/TGF-β signaling pathway (24), and enhance pulmonary hypertension and ischemia-reperfusion injury in animal models (Figure 6A). Additionally, the mechanism may be associated with the regulation of Ras homologous family member A/Rho-associated coiled-coil-containing protein kinase pathway (157, 158). Lower plasma betaine levels are associated with an increased risk of heart failure and myocardial infarction (159). In patients with hypertension, a U-shaped relationship was observed between serum betaine levels and the risk of first ischemic stroke (160). Overall, the effects of betaine on the cardiovascular system are heterogeneous across different populations and are dose-dependent. For example, daily supplementation of 4 g for more than 6 weeks may lead to a moderate increase in the levels of plasma total cholesterol (155) and trimethylamine oxide (161), which is detrimental to cardiovascular health.

### 5.5 Betaine for the prevention and treatment of kidney disease

The effects of betaine on kidney health are multifaceted across models and populations; however, these effects are context-dependent. In cats with chronic kidney disease (CKD), betaine supplementation enhances renal health and reverses weight loss, possibly through its effects on one-carbon metabolism, gut microbiota, body composition, and plasma metabolome (162, 163). Similarly, betaine positively modulated plasma and fecal metabolites in a dog model of chronic kidney disease (164). However, population-based studies have demonstrated mixed results. A study of 478 patients with CKD revealed that higher plasma betaine concentrations were a risk factor for adverse renal outcomes (165). In the context of CKD, betaine may also exhibit adverse effects, via multiple molecular mechanisms. For example, the accumulation of TMAO levels in the body promotes renal fibrosis, inflammation, and atherosclerosis, thereby accelerating the progression of CKD (166, 167); high betaine levels may lead to abnormal methylation of profibrotic genes (such as TGF-β and COL1A1), promoting renal interstitial fibrosis (168); and methylation cycle disorders in patients with CKD lead to homocysteine accumulation, whereas abnormal betaine metabolism may exacerbate reactive oxygen species production, promoting renal tubular damage (169). These abnormal changes are likely closely related to renal function, and kidney disease appears to disrupt this process (Table 1). However, plasma betaine levels were significantly associated with the estimated glomerular filtration rate and exhibited good discriminatory ability in distinguishing CKD states (170). In Chinese children and adolescents, betaine intake may reduce the risk of hyperuricemia by enhancing glomerular filtration (171). Mechanistically, numerous animal experiments have revealed the protective potential of betaine, whose effects are primarily reflected in combating oxidative stress, inhibiting inflammatory responses, and regulating primary signaling pathways. For example, in potassium chloride-induced hyperuricemic mice (172), arsenic-exposed mice (173, 174), and sodium fluoride-induced renal injury rat models (175), betaine exerts protective effects by regulating urate transporters, inhibiting oxidative stress, and modulating the Nrf-2/heme oxygenase-1 (HO-1) signaling axis (Figure 6B). In various renal injury models induced by high fructose (176), cadmium (177), cisplatin (178), doxorubicin (179), isoproterenol (180), and carbon tetrachloride (181), betaine protects renal function through various mechanisms, such as reducing lipid deposition, inhibiting inflammasome activation, and inhibiting apoptosis. Additionally, betaine enhances renal pathological damage in diabetic pregnant mice (182) and thioacetamide-induced renal injury mouse models (183).

**Table 1:** Clinical studies investigating the effects of betaine in various diseases.

Model/Population	Disease/Condition	Intervention method	Intervention dosage	Mechanism (s)	Adverse effects	References
12 healthy males; 3 patients with homocystinuria	Homocystinuria	Oral (anhydrous betaine powder)	Single dose 50 mg/kg; or 50 mg/kg twice daily	Methyl donor; converts homocysteine to methionine via BHMT enzyme	Safe and well tolerated	(13)
55 patients with biopsy-proven NASH	NASH	Oral (anhydrous betaine powder)	20 g daily for 12 months	Lowers SAH; improves hepatic steatosis	Minor GI upset in some	(106)
13,440 ARIC study participants	T2D risk	Dietary intake (prospective cohort)	Median intake: 74.5–114.8 mg/day	Osmoprotection; involvement in one-carbon metabolism	N/A (observational study)	(61)
1,292 patients with CAD	CAD mortality	Dietary intake (prospective cohort)	Range: 100–400 mg/day	Lowers SAH and tHcy levels; increases SAM and SAM/SAH ratio	N/A (observational study)	(150)
4,336 participants from the PREVEND study	T2D	Serum metabolite levels (observational)	Median plasma: 34.1 μmol/L	Regulation of lipid metabolism and insulin sensitivity	N/A (observational study)	(58)
6,022 Iranian adults	T2D	Dietary intake (prospective cohort)	Mean intake: ~104 mg/day	Potential epigenetic effects; homocysteine remethylation	N/A (observational study)	(62)
2,606 adults (Tehran Lipid and Glucose study)	CVD	Dietary intake (prospective cohort)	Geometric mean: 78 mg/day	Precursor for cell membrane phospholipids and neurotransmitters	N/A (observational study)	(146)
29,079 Japanese residents (Takayama study)	CVD	Dietary intake (prospective cohort)	Range: ~50–150 mg/day	Reduction of inflammatory markers (CRP, IL-6)	N/A (observational study)	(147)
14,430 middle-aged adults	CHD	Dietary intake (prospective cohort)	Mean: ~100 mg/day	Modification of DNA methylation and one-carbon metabolism	N/A (observational study)	(149)
Type 2 diabetes patients (ZODIAC study)	Diabetic microvascular complications	Plasma betaine levels (observational)	Circulating levels	Inhibition of VEGF signaling and mesangial cell proliferation	N/A (observational study)	(59)
8 patients with probable mild Alzheimer’s disease	AD	Oral (anhydrous powder)	3 g twice daily (6 g/day) for 24 weeks	Methyl donor; lowers serum homocysteine; increases brain methionine and SAM to delay disease progression	Diarrhea, prostatitis (1 patient); myocardial infarction (1 patient, likely unrelated)	(195)
97 AD patients (malnutrition vs. non-malnutrition)	AD; hyperhomocysteinemia	Oral (diet intervention)	50, 100, and 200 μg/kg for 1 month	Lowers Hcy; reduces tau phosphorylation; increases PP2Ac activity; inhibits Aβ accumulation; suppresses TNF-α and IL-1β	Not reported	(200)
Ischemic stroke patients (nested case–control)	Recurrent stroke; cardiovascular events	Observational (plasma levels)	N/A (baseline circulating levels)	Neurotransmitter synthesis; cell membrane integrity; methyl-group metabolism (Hcy reduction)	N/A (observational study)	(151)
Acute ischemic stroke patients (CATIS trial)	Post-stroke cognitive impairment	Observational (plasma levels)	N/A (baseline circulating levels)	Inverse association with cognitive decline; potential neuroprotective effects via one-carbon metabolism	N/A (observational study)	(152)
531 patients with ACS	Secondary MI; Heart failure; death	Observational (plasma and urine)	N/A (baseline circulating levels)	Betaine insufficiency linked to unfavorable vascular risk and metabolic syndrome profiles	N/A (observational study)	(159)
Hypertensive patients (nested case–control)	First ischemic stroke	Observational (serum levels)	N/A (baseline circulating levels)	Serves as a methyl donor; prevents premature apoptosis; osmoprotection	N/A (observational study)	(160)
3,903 stable cardiac patients undergoing angiography	MACE	Observational (plasma levels)	N/A (baseline circulating levels)	Betaine acts as a precursor to TMAO via gut microbiota; high levels are pro-atherogenic when TMAO is elevated	N/A (observational study)	(161)
Patients with moderate to advanced CKD	Cardiovascular and renal outcomes	Observational (plasma levels)	N/A (baseline circulating levels)	Interaction with renal function and gut-microbiota metabolites (TMAO); one-carbon cycle metabolism	N/A (observational study)	(165)
521 stable subjects with CKD	Chronic kidney disease; mortality	Observational (plasma levels)	N/A (baseline circulating levels)	Precursor for TMAO; contributes to renal tubulointerstitial fibrosis and progressive renal dysfunction	N/A (observational study)	(166)
179 CKD stage 3–5 patients	Inflammation and mortality in CKD	Observational (plasma levels)	N/A (baseline circulating levels)	Linked to GFR and markers of inflammation; precursor for gut-microbial generation of TMAO	N/A (observational study)	(167)
612 ischemic stroke patients (CATIS trial)	PSD	Observational (plasma levels)	N/A (circulating plasma levels)	Methyl donor; anti-inflammatory effects; neuroprotective role in neuroplasticity and recovery	N/A (observational study)	(201)
11 children with CBS or cblC deficiency	CBS deficiency; cblC deficiency (homocystinuria)	Oral (twice-daily dose)	100 mg/kg/day vs. 250 mg/kg/day	Methyl donor for homocysteine remethylation via BHMT enzyme; chemical chaperone	GI disorders (diarrhea, vomiting, pain), fever, headache, muscular pain	(202)
29 patients with CHC	CHC	Oral (combination with SAMe, PegIFN, Ribavirin)	6 g/day	Required for methionine generation; recycling of SAMe; potentiates IFNa signaling	Flu-like symptoms, fatigue, GI symptoms (diarrhea, pain), skin/mood issues	(203)
29 patients with chronic haemodialysis	ESRD; hyperhomocysteinemia	Oral (with folic acid)	4 g daily for 12 weeks	Alternate methyl donor for remethylation of homocysteine to methionine	Not specifically detailed	(204)
8 healthy white male volunteers	Elevated homocysteine (postmethionine load)	Oral (supplement or high-betaine meal)	~500 mg supplement or ~517 mg meal	Major tissue osmolyte; substrate for BHMT to remethylate homocysteine	Indigestion and diarrhea (at higher pharmacologic doses)	(205)
2,568 patients with suspected stable angina pectoris	SAP; AMI risk	Observational (plasma levels)	N/A (circulating plasma levels)	Mitochondrial oxidation product of choline; osmolyte and methyl donor	N/A (observational study)	(206)
90 patients undergoing coronary angiography	CAD; hyperhomocysteinemia	Observational/randomized (part of B-vitamin trial)	N/A (baseline/post-methionine load levels)	Substrate for BHMT; converts homocysteine to methionine; determinant of postmethionine load tHcy	Not reported	(207)
44 patients with CAD	Endothelial dysfunction; CAD	Oral (betaine anhydrous)	100 mg/kg/day for 6 weeks	Homocysteine lowering via remethylation; testing effect on FMD	Not reported	(208)
Patients with SBS	Hepatopathy (hepatic steatosis)	Oral (betaine anhydrous)	10 g daily (divided into 2 doses) for 3 months	Decreases hepatic fat; anti-inflammatory (reduces IL-6, TNF-α); homocysteine regulation	Well-tolerated; no significant adverse effects	(209)
5 patients with pyridoxine-non-responsive homocystinuria	Homocystinuria; Osteoporosis (low bone density)	Oral (crossover study)	3 g twice daily (6 g/day) for 2 years	Reduces plasma homocystine; increases plasma methionine	No adverse effects	(210)
161 postmenopausal women	Cardiovascular risk; homocysteine status	Oral (choline bitartrate as betaine precursor)	400 mg/day or 1,100 mg/day choline	Choline is oxidized to betaine; betaine acts as a methyl donor to lower fasting tHcy	Not reported	(211)
Animal models (implied through metabolic pathways) and humans	Atherosclerosis and CKD	Dietary intake/metabolism as a precursor to TMAO	Not specified (noted as a “lesser degree” contributor to TMAO compared to choline or L-carnitine)	Choline oxidation produces high levels of betaine, which is converted into TMAO by gut microbiota, increasing the risk of CKD	Increased TMAO leading to renal tubulointerstitial fibrosis and dysfunction	(166)

ACS, acute coronary syndrome; AD, Alzheimer’s disease; ARIC, atherosclerosis risk in communities; CAD, coronary artery disease; CATIS, China Antihypertensive Trial in Acute Ischemic Stroke; CHC, chronic hepatitis C; CHD, coronary heart disease; CKD, chronic kidney disease; CVD, cardiovascular disease; ESRD, end-stage renal disease; eGFR, estimated glomerular filtration rate; FMD, flow-mediated dilatation; Hcy, homocysteine; NASH, nonalcoholic steatohepatitis; PREVEND, prevention of renal and vascular end-stage disease; PSD, poststroke depression; SAH, S-adenosylhomocysteine; SAMe, S-adenosyl-methionine; SAP, stable angina pectoris; SBS, short bowel syndrome; T2D, type 2 diabetes; TMAO, trimethylamine-N-oxide; UACR, urine albumin-to-creatinine ratio; UAE, urinary albumin excretion.

### 5.6 Betaine for the prevention and treatment of Alzheimer’s disease

Betaine has demonstrated multipathway protective potential in various experimental models of Alzheimer’s disease (AD). In amyloid-beta (Aβ)-induced AD rat or mouse models, betaine can effectively enhance cognitive dysfunction and memory impairment, and its effects are closely associated with increasing GSH, reducing oxidative markers such as MDA, and activating primary proteins such as SIRT1 (184–186). In a homocysteine-induced cognitive impairment model, an independent risk factor for AD, betaine alleviated cognitive impairment by inhibiting the NLRP3/caspase-1/GSDMD pathway in an m^6^A-YTHDF2-dependent manner, thereby suppressing microglial pyroptosis (187). At the molecular level, betaine regulates the processing of amyloid precursor proteins by enhancing *α*-secretase activity and reducing β-secretase activity, thereby reducing Aβ production (188). Additionally, it activates PP2A to reduce Tau protein hyperphosphorylation (189) and inhibits the nucleotide-binding oligomerization domain-, leucine-rich repeat- and pyrin domain-containing protein 3/NF-κB signaling pathway to alleviate neuroinflammation (190) (Figure 6C). Moreover, betaine exerts neuroprotective effects by inhibiting the expression of ferroptosis-related factors (acyl-CoA synthetase long-chain family member 4 and transferrin receptor 1) (191). Furthermore, in 3xTg transgenic AD model mice and *Caenorhabditis elegans* models, betaine enhanced synaptic function and alleviated Aβ toxicity (192, 193). In clinical research, a meta-analysis of 7,009 patients with AD indicated that betaine enhanced the cognitive function and improved the quality of life (194). However, the results of small-scale human trials remain unclear and should be verified with larger samples (195). In summary, betaine may play a significant role in the prevention and intervention of AD through various mechanisms, such as anti-oxidation, anti-inflammation, regulation of Aβ and Tau pathology, and inhibition of ferroptosis.

## 6 Challenges for the use of betaine in disease prevention and treatment

Based on the current research on betaine use for the prevention and treatment of chronic diseases (196), the vast majority of experimental results from basic research have been positive. However, disease enhancement has rarely been observed in clinical studies. This may be because numerous factors affect the population and studies primarily rely on semi-quantitative questionnaires that limit the ability to accurately assess intake. Additionally, betaine is easily absorbed by the human body and its intake is not problematic. Betaine primarily acts as a methyl donor; however, folic acid, vitamin B12, and choline can also serve this function, which may diminish its unique molecular biological role. The mechanisms of betaine metabolism likely involve methylation, gut microbiota, inflammation, oxidative stress, and apoptosis. Owing to its complexity, betaine may be more suitable for dietary supplementation rather than for targeted drug development. High betaine intake may cause gastrointestinal discomfort, and the long-term safety of high-dose use remains limited, particularly in patients with chronic diseases or in the elderly. During normal supplementation, a daily intake of no more than 6 mg/(kg·bw) of betaine is considered safe (197). High doses (≥3 g/single dose) can cause nausea, diarrhea, and gastrointestinal discomfort, possibly due to increased intestinal water secretion caused by high osmotic pressure. These dose-dependent discomfort symptoms can be alleviated by taking the medication in divided doses or with food. Betaine should be used with caution in individuals with CKD and in those requiring cholesterol control, as it may exacerbate disease progression (166, 198). Current research also indicates that betaine has extremely low toxicity and is not heritable (197, 199). Therefore, large-scale clinical research and deeper mechanistic exploration are required to supplement its specific effects, supplementary dosage, and disease intervention time to maximize its role in preventing and treating chronic diseases.

## 7 Conclusion

As a primary methyl donor and osmotic pressure regulator, betaine plays a crucial role in the prevention and treatment of various chronic diseases (obesity, diabetes, metabolism-related fatty liver disease, CKD, and Alzheimer’s disease). Its mechanism of action primarily involves inhibiting the inflammatory response, reducing oxidative stress, resisting apoptosis, and regulating gene expression through epigenetic modifications (DNA methylation). Additionally, betaine can significantly alleviate tissue damage and disease processes caused by drug toxicity, viral infection, and chemical exposure. Although preclinical research data on betaine are encouraging, the conclusions drawn from population epidemiological surveys and randomized controlled clinical trials are inconsistent, and most studies have failed to its replicate significant protective effects. This indicates that the therapeutic efficacy of betaine may be affected by various complex factors. However, in this article, we did not compare all the benefits of betaine and exercise and only analyzed the main mechanisms by which exercise affects disease prevention and treatment. Furthermore, it lacks a comprehensive and detailed analysis of the molecular mechanisms of betaine, as well as quantitative data presentation. Future research should introduce advanced technologies, such as multiomics integrated analysis to systematically clarify the precise targets and core regulatory networks of betaine in the human body, reveal the reasons for its variable effects in different individuals, and identify the subgroups most likely to benefit. Therefore, these in-depth studies will provide primary evidence for determining whether betaine has the potential to be a clinically effective nutritional supplement or therapeutic drug and guide its precise application.
